# Activating Transcription Factor 3 Protects against Restraint Stress-Induced Gastrointestinal Injury in Mice

**DOI:** 10.3390/cells10123530

**Published:** 2021-12-14

**Authors:** Dun-Jie Chuang, Subhashree Pethaperumal, Bijaya Siwakoti, Hung-Jen Chien, Ching-Feng Cheng, Shih-Che Hung, Te-Sheng Lien, Der-Shan Sun, Hsin-Hou Chang

**Affiliations:** 1 Department of Molecular Biology and Human Genetics, Tzu-Chi University, Hualien 970, Taiwan; 104727107@gms.tcu.edu.tw (D.-J.C.); subhashreepethaperumal@gmail.com (S.P.); bijaya2580@gmail.com (B.S.); alan211@mail.tcu.edu.tw (T.-S.L.); dssun@mail.tcu.edu.tw (D.-S.S.); 2 Institute of Biotechnology, National Tsing Hua University, Hsinchu 300, Taiwan; chienhj1026@gmail.com; 3 Department of Pediatrics, Taipei Tzu Chi Hospital, Buddhist Tzu Chi Medical Foundation, New Taipei City 231, Taiwan; chengcf@mail.tcu.edu.tw; 4 Institute of Biomedical Sciences, Academia Sinica, Taipei 115, Taiwan; 5 Institute of Medical Sciences, Tzu-Chi University, Hualien 970, Taiwan; 102353113@gms.tcu.edu.tw

**Keywords:** ATF3, gastrointestinal tract, intestinal leakage, tight junction, cell death, gut epithelial cell, stress ulcer

## Abstract

Psychological stress increases the risk of gastrointestinal (GI) tract diseases, which involve bidirectional communication of the GI and nerves systems. Acute stress leads to GI ulcers; however, the mechanism of the native cellular protection pathway, which safeguards tissue integrality and maintains GI homeostasis, remains to be investigated. In a mouse model of this study, restraint stress induced GI leakage, abnormal tight junction protein expression, and cell death of gut epithelial cells. The expression of activating transcription factor 3 (ATF3), a stress-responsive transcription factor, is upregulated in the GI tissues of stressed animals. ATF3-deficient mice displayed an exacerbated phenotype of GI injuries. These results suggested that, in response to stress, ATF3 is part of the native cellular protective pathway in the GI system, which could be a molecular target for managing psychological stress-induced GI tract diseases.

## 1. Introduction

Psychological stress can increase the risk of gastrointestinal (GI) tract diseases [1,2,3]. Major mental disorders [4], such as depression and anxiety [2], bipolar disorder [5], schizophrenia [6], dementia [7,8], and autism [9], are all associated with an increased risk of GI tract diseases. Such a high correlation between psychological stress and GI tract disorders may be explained through bidirectional communication of the gut–brain axis that is composed of the central nervous system (CNS) and neuroendocrine, immune, and GI systems [10,11]. GI tract defects may increase the risk of and exacerbate CNS disorders [7,8]. These GI tract disorders are developed chronically; however, the short-term impact of psychological stress on the development of GI tract disorders remains elusive. Additionally, from a therapeutic point of view, methods of diagnosing and detecting early GI tract disorders are required.

Activating transcription factor 3 (ATF3), a member of the mammalian activation transcription factor/cAMP responsive element-binding protein family, is a stress-induced transcription factor critical in modulating metabolism, immunity, and oncogenesis [12]. Several pieces of evidence have indicated that ATF3 plays a critical role in regulating the homeostasis of the GI system. For example, ATF3 is critical in maintaining mucosal immunity [13] and healthy immune-microbiota interactions [14]. ATF3 also ameliorates colitis [15] and facilitates intestinal regeneration [16]. This evidence collectively suggests that ATF3 plays a crucial role in the maintenance of normal intestinal function. However, the role of ATF3 in acute psychological stress-induced GI injuries remains elusive.

Examining restraint stress is a commonly used method to investigate acute or chronic psychological stress-related behavioral, biochemical, and physiological changes in laboratory animals [17,18,19]. After experiencing restraint and immobilization stress, animals displayed higher levels of anxiety [17]. Chronic restraint stress stimulations that last for weeks can lead to brain abnormalities [20,21,22], intestinal dysfunction [23], and dysbiosis [24,25,26] in experimental animals. Since the gut–brain axis is a bidirectional regulation system between the CNS and GI tract [27], restraint stress can affect both sides of the gut–brain axis, and using the restraint stress model is a feasible means for the development of methods of early diagnosis and detection of stress-induced GI disorders. However, measurement methods of gut leakage in the currently available animal models present several disadvantages. For example, the methods may be time-consuming (e.g., the lactulose/mannitol test requires two orally administered nonmetabolized sugars over a 6-h period) or require specific equipment (e.g., high-pressure liquid chromatography or liquid chromatography combined with mass spectrometry) or isotope labeling (e.g., ^51^Cr-ethylenediaminetetraacetic acid) [28]. Endoscopy examination is useful on the evaluation and management of duodenal ulcer in clinical settings [29,30]. However, because mice are too small, such technique is not feasible in this model, despite how we still see some hemorrhage lesions in the GI tract of stressed mice (Figure S1A). As a result, to observe timely results of gut leakage in the experimental animal, an easier method is desired.

We developed a simple method to analyze stress-induced GI leakage. Using intestinal, nonabsorbable, Evans blue dye, we observed the timely changes of mouse intestinal leakage. We used a combination of analysis methods, namely flow cytometry, quantitative reverse transcription polymerase chain reaction (qRT-PCR), and immunohistochemistry (IHC) staining analyses, to analyze the cellular and molecular changes in the early phase of the injury. Restraint stress induced an ulcer-like injury in mice, and proton pump inhibitor (PPI) treatments rescued the gut leakage. Additionally, an ATF3 deficiency exacerbated the leakage in mice, suggesting that ATF3 plays a protective role in stress ulcers.

## 2. Materials and Methods

### 2.1. Laboratory Mice

Wild-type (WT) C57BL/6J mice aged 8–12 weeks were purchased from the National Laboratory Animal Center (Taipei, Taiwan) [31,32,33,34,35,36]. Genetically deficient *ATF3*^−/−^ (ATF3 KO) mice with a C57BL/6J background [37,38] were provided by Dr. Tsonwin Hai. *ATF3*^−/−^ mice were backcrossed with WT C57Bl/6J mice over six generations. All animals were housed in the Animal Center of Tzu-Chi University in a specific pathogen-free temperature- and light-controlled environment with free access to filtered water and food. Approximately 300 wild-type mice and 80 *ATF3*^−/−^ mice were employed in this study. All experimental protocols for examining the experimental animals were approved by the Animal Care and Use Committee of Tzu-Chi University, Hualien, Taiwan (approval ID: 110024).

### 2.2. Measurement of Stress-Induced GI Leakage

To induce restraint stress [39,40], the mice were kept in a 50-mL plastic falcon tube for 9 h. Holes were created at the tapering end of the falcon tube to ensure sufficient air supply. Blood samples (50 µL) were collected four times during the experiment at 0, 5, 7, and 9 h after the stress challenge began. Evans blue (1.2 g/kg, Santa Cruz Biotechnology, Santa Cruz, CA, USA) was fed to the mice using a steel feeding tube 4 h after the commencement of stress challenge. Their blood plasma was isolated by collecting blood in an Eppendorf tube and mixing it with an equal proportion of anticoagulant citrate dextrose solution to prevent coagulation [41,42,43]. The collected plasma was transferred to 96-well plates, in which the concentration of Evans blue was determined using a full-spectrum analyzer (Multiskan Spectrum, Thermo Fisher Scientific, Waltham, MA, USA) at 620 nm.

### 2.3. qRT-PCR Analysis

#### 2.3.1. RNA Isolation and cDNA Preparation

After 9 h of stress, a duodenum sample (1 cm; begins immediately after the pyloric portion of the stomach) from the mice was isolated, washed (phosphate-buffered saline, PBS) and dissolved in Trizol (Ambion, Thermo Fisher Scientific) reagent. After the implementation of the ribonuclease-free deoxyribonuclease treatments and standard isolation protocols, the RNA concentration was analyzed using a NanoDrop spectrophotometer (Thermo Fisher Scientific). The RNA (1 μg) was used to synthesize complementary DNA (cDNA) using an iScript cDNA Synthesis Kit (Bio-Rad Laboratories, Hercules, CA, USA). The obtained cDNA was used for PCR and qRT-PCR analysis, and the samples were stored at −20 °C before use.

#### 2.3.2. qRT-PCR

We used qRT-PCR to analyze the expression of tight junction genes, such as zonula occludens-1 (ZO-1), claudin 3 (CLDN3), and junctional adhesion molecule 3 (JAM3), molecules involve in the maintenance of mucosal homeostasis and intestinal integrality [44,45,46,47], in the stomach, duodenum, and jejunum, respectively. cDNA (2 µL) was mixed with 10-µL SYBR Green (Thermo Fisher Scientific), 0.5 µL each of forward and reverse primers, and 7-µL ddH_2_O and quantified in a real-time reverse transcription linkage instrument (StepOnePlus Real-Time PCR System, Thermo Fisher Scientific) with varying annealing temperatures according to the primers. Each sample was run in triplicate, and the average cycle threshold (Ct) values were used to calculate 2^−ΔΔCT^ with that of the internal control—GAPDH (glyceraldehyde-3-phosphate dehydrogenase) gene expression.

### 2.4. Flow Cytometry Analysis

After 9 h of stress, the duodenum samples (6 cm, begins immediately after the pyloric portion of the stomach; PBS-washed) were cut into tiny pieces and incubated with a collagenase D (Sigma-Aldrich, Burlington, MA, USA; 1 mg/mL)-containing serum-free cell culture medium for 30 min in a 15-mL falcon tube at 37 °C with shaking (upright shaking incubator; OSI 500, Kansin Instruments, New Taipei City, Taiwan). After the removal of the media by centrifugation, mouse GI epithelial cells were dissociated from the remaining cell and tissue pellets by incubation with 2 mL of nonenzymatic cell dissociation solution (Sigma-Aldrich) for 10 min at room temperature. After being washed, each sample of the dissociated cells was fixed with 500 µL of a fixation buffer (Cytofix, BD Biosciences, San Jose, CA, USA), mixed properly, and incubated at room temperature for 20 min. These samples were then centrifuged at 300× *g* for 5 min. After washing (Perm/Wash buffer, BD Biosciences) and blocking (5% bovine serum albumin in RPMI), the cells were subjected to the staining of CD326 (a gut epithelial cell marker [48,49]), tight junction, and cell death marker (anti-CD326 epithelial cell marker antibody, BioLegend; anti-occludin antibody and anti-claudin-3 antibody, Thermo Fisher Scientific; anti-cleaved caspase-3 antibody, Cell Signaling Technology, Danvers, MA, USA; propidium iodide (PI), Thermo Fisher Scientific; and annexin V, Thermo Fisher Scientific). The samples were then analyzed through flow cytometry (Gallios, Beckman Coulter Life Sciences, Brea, CA, USA) for the quantitative analysis [50,51,52].

### 2.5. Immunohistochemistry (IHC) Tissue Section Preparation

After 9 h of stress, the duodenum samples (6 cm; begins immediately after the pyloric portion of the stomach, PBS-washed) were collected and rinsed with 1 mL of ice-cold PBS 3 times and Bouin’s reagent (1 mL, 50% ddH_2_O, 45% absolute ethanol, and 5% acetic acid) 2 times through the opening of the duodenum. The duodenum was then cut open longitudinally using a surgical blade and washed an additional 2 times with PBS. It was then bisected along its length and Swiss-rolled with a toothpick [53]. Once the entire length of the intestine had been rolled, a pair of forceps was used to remove the rolled tissue from the toothpick, and the tissue was kept in a tissue-embedding cassette in 10% buffered formalin for more than 30 min for fixation. The rolled tissue was then placed in the organic solvents overnight for dehydration. The dehydrated tissue was then placed in a metal cassette and filled with hot wax. It was cooled until the wax solidified, and the metal cassette was removed. Following the standard protocols of paraffin embedding, removal, antigen retrieval, washes (TBS buffer), and protein blocking (5% BSA) were conducted [33,54], and the sectioned tissues were subjected to immunofluorescent staining.

### 2.6. Immunofluorescent Staining

Following the previously described methods [33,54], the tissue sections were treated with antibodies diluted with 1% BSA prepared in a TBS buffer. An anti-occludin antibody (Thermo Fisher Scientific), anti-claudin-3 antibody (Thermo Fisher Scientific), anti-cleaved caspase-3 antibody (Cell Signaling Technology), and 4′,6-diamidino-2-phenylindole (DAPI; Sigma-Aldrich) were used for staining. After staining, cover slips were mounted with 30-µL mowiol 4–88 mounting solution (2.5% 1,4-diazabicyclo-octane, 10% Mowiol 4–88, 25% glycerol, and 0.1-M Tris-HCl).

### 2.7. Imaging of Confocal Microscopy

Fluorescence imaging was conducted with confocal microscopes (LSM 800, ZEN 2.1 software, Carl Zeiss, Jena, Germany). The Zeiss confocal microscope is equipped with Plan-Apochromat 10×/0.45, Plan-Apochromat 20×/0.8, and Plan-Apochromat 40×/1.3 Oil DIC (UV) VIS-IR lenses. The 10× lens was applied to capture the gross view (under tile scan mode) and then automatically stitched by the software. The 20× and 40× lenses were used to capture detailed structures of the specimens. All scanning was performed under the multichannel mode to obtain sequential signals; the illumination power was identical for each slide. The image contrast was also optimized, and a despeckle filter was used. The DAPI-labeled nuclei were excited using a 405-nm laser, and the signals were collected with an SP 470 filter. The green fluorescence-labeled structures were excited using a 488-nm laser, and the fluorescence signal was collected with an SP 545 filter. The red fluorescence-labeled structures were excited using a 543-nm laser, and the signals were collected with an SP 620 filter. An analysis of at least three independent images was conducted in one section, and quantified results of the fluorescence intensity were obtained using ImageJ software (1.52a, National Institutes of Health, Bethesda, MD, USA) [33,42].

### 2.8. Statistical Analyses

The experimental results were analyzed using Microsoft Office Excel 2003 (Redmond, WA, USA) and SPSS 17 (International Business Machines Corporation, Armonk, NY, USA), and the results were reported as mean ± standard deviation. Statistical significance of the obtained results was examined using a one-way analysis of variance and post hoc Bonferroni-corrected *t*-test; a probability of type 1 error α = 0.05 was considered the threshold of statistical significance. Following a key principle governing the ethical use of animals in research that no animal life is wasted, the number of animals used in each experiment must be the minimum necessary to obtain valid and meaningful results [55,56], so we set the level of significance/alpha/type I error of our experiment data at 5% (0.05) and the power/beta/type II error at 80% [55] to test our hypothesis. Software G*Power 3.1.9.2 was employed to calculate the mouse sample size needed [57,58]. For example, to compare the mean difference of the plasma Evans blue levels between the 2 groups (WT no stress and WT stress) using G*Power, we determined the effect size as 2.25. If we further set α as 0.05 and the power(1-β) as 0.80, in a two-sided test, we then obtained an estimated total sample size of 10 in 2 groups (i.e., 5 per group, *n* = 5). To compare the mean differences of plasma Evans blue levels among the 4 groups (WT no stress, WT stress, ATF3 KO no stress, and ATF3 KO stress), according to the G*Power analyses, we set an effect size of 1.23, α of 0.05, power(1-β) of 0.80, and a two-sided test, and then, we obtained an estimated total sample size of 12 in 4 groups (i.e., 3 per group, *n* = 3). Thus, a total of 3–6 mice per group were considered necessary in this study based on the data analyses of the individual experiments.

## 3. Results

### 3.1. Restraint Stress-Induced GI Tract Leakage Is Sensitively Revealed through Measurement of Plasma Evans Blue Levels That Leaked from GI after Gavage of Live Mice

**Figure 1:**
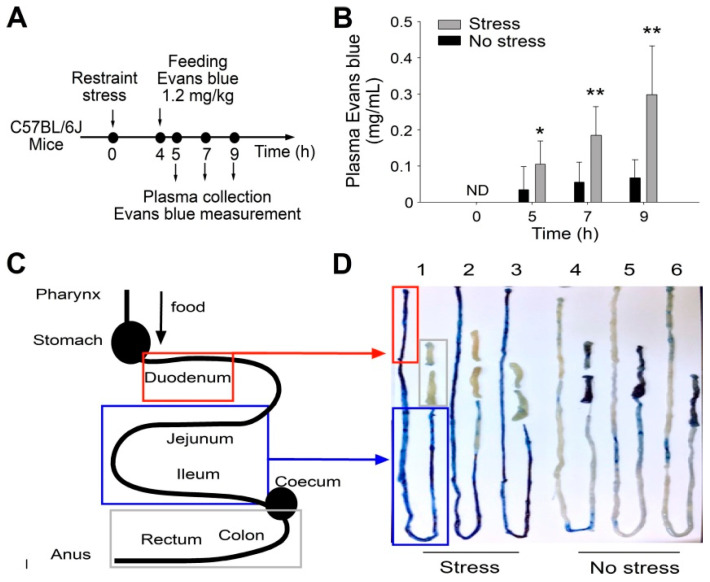
Restraint stress-induced GI leakage is revealed by GI administration of Evans blue. (**A**) Experiment outline of the restraint stress mouse model with Evans blue treatments. (**B**) Plasma Evans blue levels of mice with or without restraint stress at 0, 5, 7, and 9 h. ND: not detected. Error bars show the standard deviation; * *p* < 0.05 and ** *p* < 0.01 vs. their respective no stress groups. (**C**) A schematic diagram of the mouse GI system according to the information provided by previous reports [59,60]. (**D**) Residue Evans blue in the mouse GI system after the experiment. The residue Evans blue primarily localized in the small intestine (duodenum and jejunum) region in the stressed groups. *n* = 9 (3 experiments with a total of 9 mice per group).

In this study, orally administrated Evans blue was markedly increased in the plasma of those live mice under restraint stress but not in the control groups without stress. The experiment outline and plasma Evans blue levels are presented in Figure 1A,B, respectively. An examination of the intestinal tissues revealed that, compared with the control groups, Evans blue was primarily distributed in the small intestine of the stressed mice (Figure 1C,D). This suggests that the small intestine is a leakage site after restraint stress.

**Figure 2:**
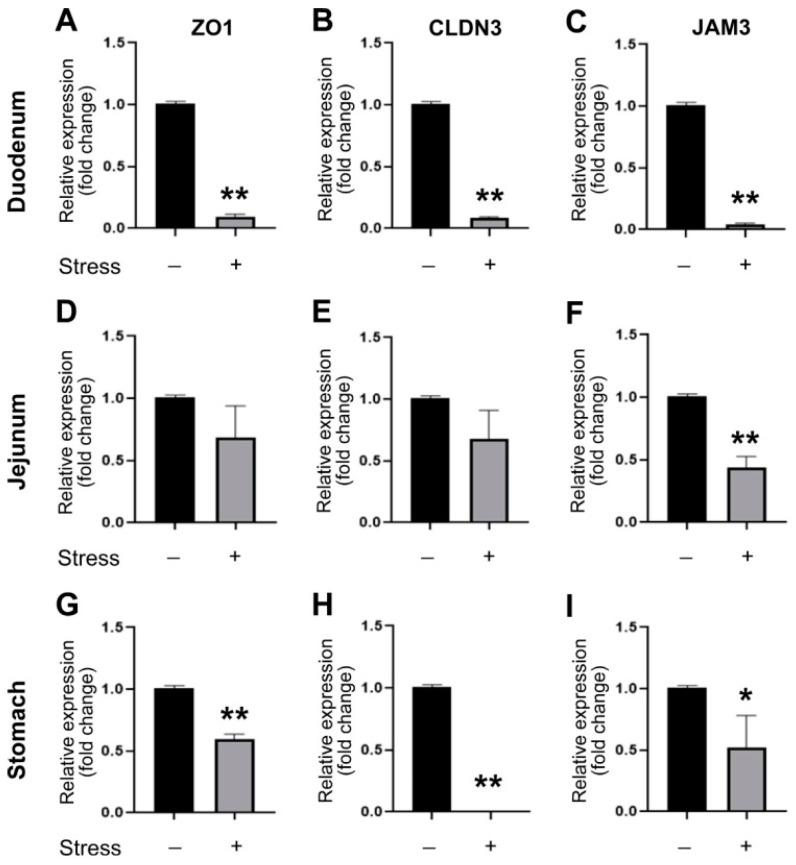
Relative mRNA expression levels of tight junction genes. Through qRT-PCR analysis, the relative mRNA expression levels of tight junction genes ZO-1 (**A**,**D**,**G**), CLDN3 (**B**,**E**,**H**), and JAM3 (**C**,**F**,**I**) and of the mouse duodenum (**A**–**C**), jejunum (**D**–**F**), and stomach (**G**–**I**) with or without 9 h of restraint stress. The mRNA expression levels of the control (no stress) groups were normalized to 1-fold. *n* = 3 (2 experiments with a total of 3 mice per group). * *p* < 0.05 and ** *p* < 0.01 vs. their respective no stress control groups. The “stress −“ indicates no stress control groups and the “stress +” indicates the stressed groups.

The expressions of these tight junction genes are highly affected in the duodenum and stomach and less affected in the jejunum (Figure 2; duodenum and stomach with suppression up to a total of three out of three tested tight junction genes; jejunum with one-third). In addition, the mRNA expression levels of the tight junctions appeared more severely affected in the duodenum than in the stomach (Figure 2), suggesting a relatively higher impact of restraint stress in the duodenal region.

### 3.2. Rescued by PPI Treatments, Restraint Stress-Induced GI Leakage Involves an Ulcer-like Injury

**Figure 3:**
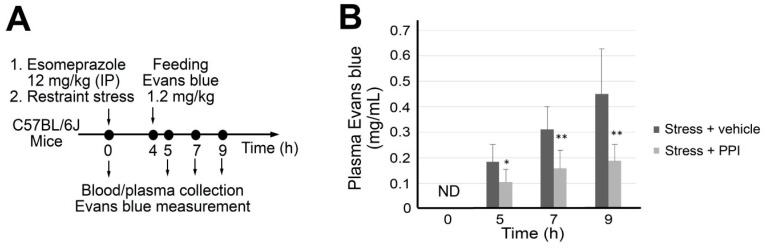
Proton pump inhibitor (PPI) rescues stress-induced GI leakage. (**A**) Experiment outline of PPI esomeprazole treatments in the restraint stress mouse model. (**B**) Plasma Evans blue levels of stressed mice with or without esomeprazole treatments at 0, 5, 7, and 9 h. Error bars show the standard deviation; * *p* < 0.05 and ** *p* < 0.01 vs. their respective control groups without esomeprazole treatments. ND: not detected. *n* = 6 (3 experiments with a total of 6 mice per group).

Duodenal ulcer-like pathophysiology is further supported by the results of the pH analysis and PPI treatments. Restraint stress markedly induced a more acidic condition in the duodenum than in the jejunum (Figure S1B). This is consistent with the qRT-PCR analyses (Figure 2) that the duodenum is a severely affected region. Additionally, PPI esomeprazole treatments in mice markedly ameliorated the restraint stress-induced gut leakage (Figure 3). These results suggested that a duodenal ulcer-like injury is involved in the restraint stress-induced GI tract leakage.

### 3.3. ATF3 Deficiency Exacerbated Stress-Induced Gut Leakage and Disrupted mRNA of Tight Junction Protein ZO-1, CLDN3, and JAM3 Expression in Mouse Duodenum

**Figure 4:**
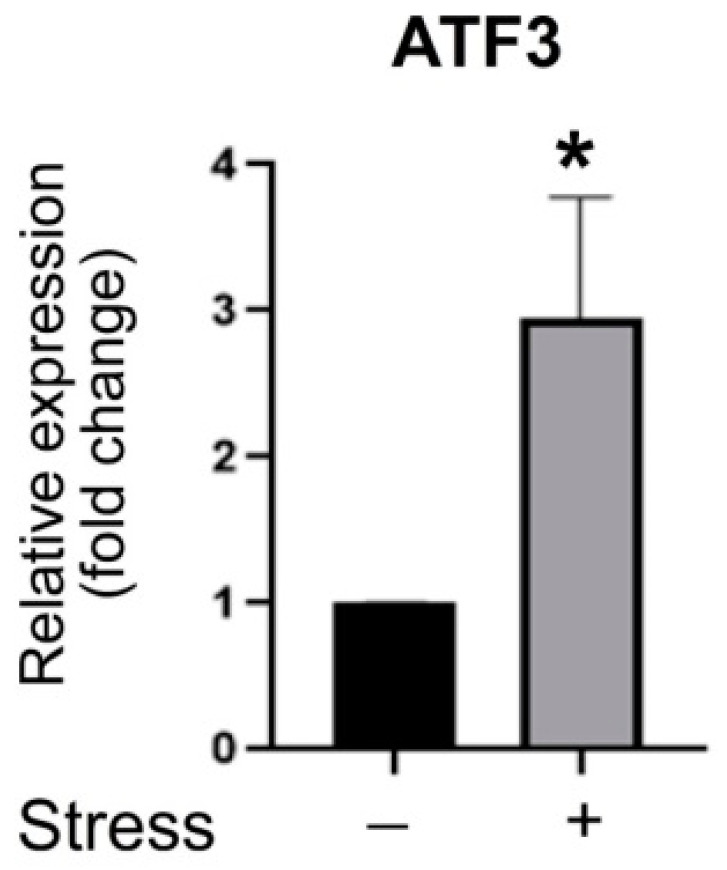
Relative duodenal mRNA expression levels of the *ATF3* gene in WT mice. Through qRT-PCR analysis, the relative mRNA expression levels of the ATF gene of the mouse duodenum with or without 9 h of restraint stress. The mRNA expression levels of the control (no stress; stress−) groups were normalized to 1-fold. Error bars show the standard deviation. *n* = 3 (2 experiments with a total of 3 mice per group). * *p* < 0.05 vs. no stress control groups. The “stress −“ indicates no stress control groups and the “stress +” indicates the stressed groups.

**Figure 5:**
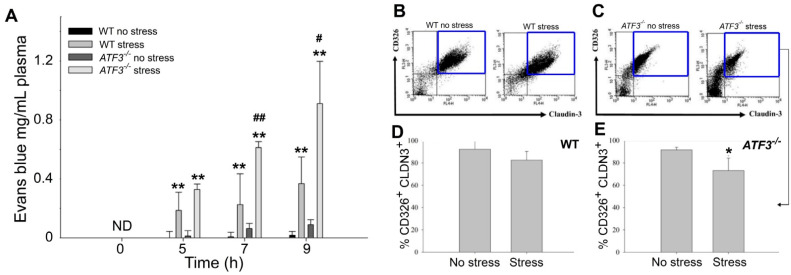
An ATF3 deficiency exacerbates stress-induced GI injuries and claudin 3 (CLDN3) suppression. (**A**) Plasma Evans blue levels of WT (WT) and ATF3 gene knockout (KO; *ATF3^−/^^−^*) mice with or without stress at 0, 5, 7, and 9 h. (**B**,**C**) Flow cytometry analysis of tight junction protein CLDN3 expressing gut epithelial (CD326^+^/CLDN3^+^ double positive) cells in WT (**B**) and *ATF3^−/−^* (**C**) mice. (**D**,**E**) The quantitative results of flow cytometry CD326^+^/CLDN3^+^ populations in WT (**D**) and *ATF3^−/^^−^* (**E**) mice are indicated. (**A**) # *p* < 0.05 and ## *p* < 0.01 vs. their respective WT groups and (**A**–**E**) * *p* < 0.05, ** *p* < 0.01 vs. no stress groups; error bars show the standard deviation. (**A**) *n* = 6 (3 experiments with a total of 6 mice per group) and (**B**–**E**) *n* = 4 (2 experiments with a total of 4 mice per group).

**Figure 6:**
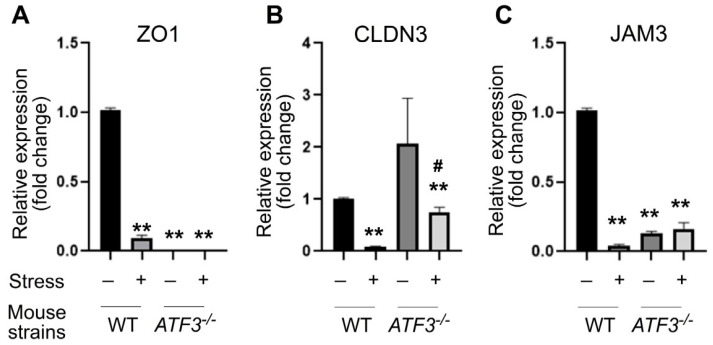
Relative duodenal mRNA expression levels of tight junction genes in WT and *ATF3^−/−^* mice. Through a qRT-PCR analysis, the relative mRNA expression levels of tight junction genes ZO-1 (**A**), CLDN3 (**B**), and JAM3 (**C**) of the mouse duodenum with or without 9 h of restraint stress. The mRNA expression levels of the control (no stress; stress-) groups were normalized to 1-fold. *n* = 3 (2 experiments with a total of 3 mice per group). Error bars show the standard deviation; ** *p* < 0.01 vs. the respective no stress WT control groups and # *p* < 0.05 vs. the respective no stress *ATF3^−/−^* control groups. The “stress −“ indicates no stress control groups and the “stress +” indicates the stressed groups.

**Figure 7:**
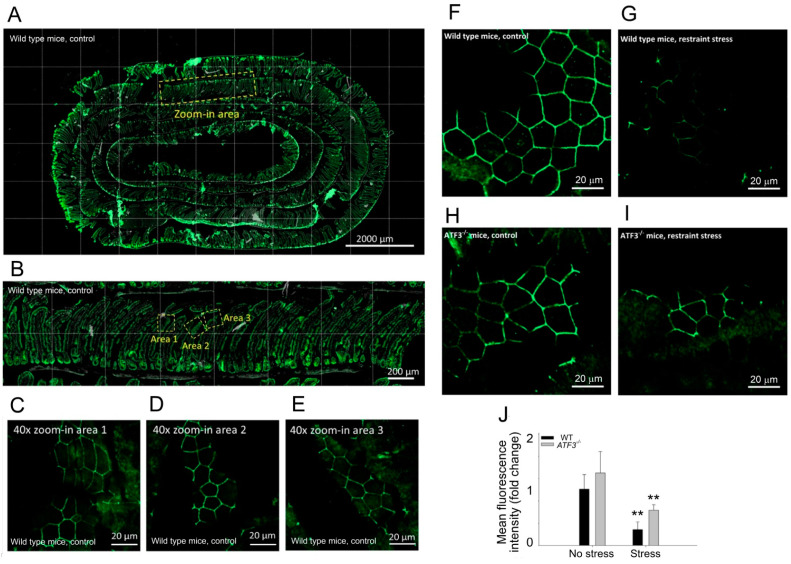
Confocal microscopy of immunohistochemistry (IHC) CLDN3 staining. Tissue section IHC of a Swiss-rolled duodenal sample was stained with a fluorescence-labeled anti-CLDN3 antibody. (**A**) A representative image at low magnification is presented. (**B**) The yellow boxed zoomed-in area in (**A**) is enlarged as a higher magnification view. (**C**–**E**) Areas 1, 2, and 3 in (**B**) are further enlarged to observe the detailed expression patterns of CLDN3 in the villi of the duodenal tissue. (**F**–**I**) The CLDN3 staining in the WT mice (**F**,**G**) was compared to *ATF3^−/−^* mutants (**H**,**I**) without (**F**,**H**) or with (**G**,**I**) restraint stress. (**J**) The quantitative results are obtained from 3 images at various areas; ** *p* < 0.01 vs. the respective no stress groups. (**J**) The averaged mean fluorescence intensity of the WT no stress groups was normalized to 1-fold. (**A**–**G**) WT mice and (**H**,**I**) *ATF3^−/−^* mutants. Scale bars: 2000 μm (**A**), 200 μm (**B**), and 20 μm (**C**–**I**). Error bars show the standard deviation.

Since the duodenum is highly affected by stress, we used duodenal tissue as a putative injury tissue to perform subsequent experiments. The analysis results of qRT-PCR further revealed that the messenger RNA (mRNA) of ATF3 is upregulated by restraint stress in the duodenal tissue (Figure 4). Since ATF3 is a stress response regulator, it plays a protective role in various diseases; ATF3 gene knockout (*ATF3^−/^^−−/^^−^* KO) mice were compared to the WT mice to examine the role of ATF3 in this system. An ATF3 deficiency markedly exacerbated restraint stress-induced gut leakage (Figure 5A), indicating a protective role of ATF3 in this system. Analyses of duodenal tissue with qPT-PCR revealed that, compared with the WT mice, the mRNA expression levels of tight junction ZO-1 and JAM3 were largely suppressed in *ATF3^−/^^−^* mice, with and without stress (Figure 6). CLDN3 mRNA was markedly suppressed after stress in the WT and *ATF3^−/^^−^* mice (Figure 6). These results suggest that, under an ATF3-deficient condition (e.g., *ATF3^−/^^−^* mice), the mRNA of the tight junction genes is not properly regulated. The flow cytometry results were consistent with the qPT-PCR data (Figure 6) and further indicated that the tight junction protein CLDN3 tended to be suppressed in the CD326^+^ duodenal epithelial cells in both WT and *ATF3^−/−^* mice (Figure 5B–E). Notably, in addition to the reduced CLDN3 levels, the CD326 levels of the duodenal cells also seem reduced in ATF3-deficient mice with an unknown mechanism (Figure 5B vs. Figure 5C; reduced CD326^+^ population in *ATF3^−/^^−^* mice). IHC data also revealed that CLDN3 was markedly suppressed in WT and *ATF3^−/−^* mice after stress (Figure 7). These results collectively suggest that a dysregulated tight junction is involved in stress-induced intestinal changes.

### 3.4. ATF3 Deficiency Exacerbated Stress-Induced Cell Death of Duodenal Epithelial Cells

**Figure 8:**
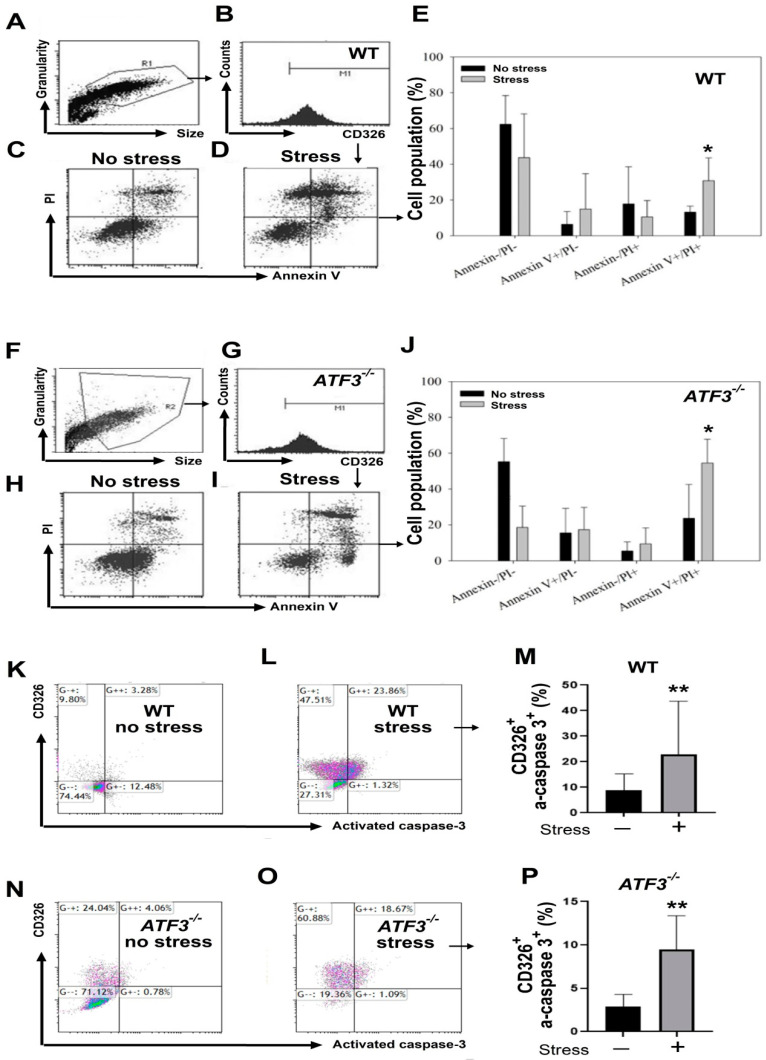
Flow cytometry results of restraint stress-induced GI cell death. (**A**–**E**) Analyses of WT mouse samples. The gating (**A**,**B**) and analysis (**C**,**D**) of CD326^+^/PI^+^/annexin V^+^ dead duodenal epithelial cells from mice without (**C**) or with (**D**) restraint stress, and the quantitative results (**E**) are revealed. (**F**–**J**) Analyses of the *ATF3^−/^^−^* mouse samples. The gating (**F**,**G**) and analysis (**H**,**I**) of CD326^+^/PI^+^/annexin V^+^ dead duodenal epithelial cells from mice without (**H**) or with (**I**) restraint stress, and the quantitative results (**J**) are indicated. (**K**–**P**) Relative levels of CD326^+^/activated-form caspase-3^+^ (a-caspase 3^+^) apoptotic epithelial cells in WT (**K**–**M**) and *ATF3^−/^^−^* (**N**–**P**) mice with or without stress are revealed (gating in (**K**,**L**,**N**,**O**)). (**M**,**P**) The quantitative results are indicated. *n* = 5 (2 experiments with a total of 5 mice per group). (**E**,**J**,**M**,**P**) * *p* < 0.05 and ** *p* < 0.01 vs. their respective no stress control groups. Error bars show the standard deviation. The “stress −“ indicates no stress control groups and the “stress +” indicates the stressed groups.

**Figure 9:**
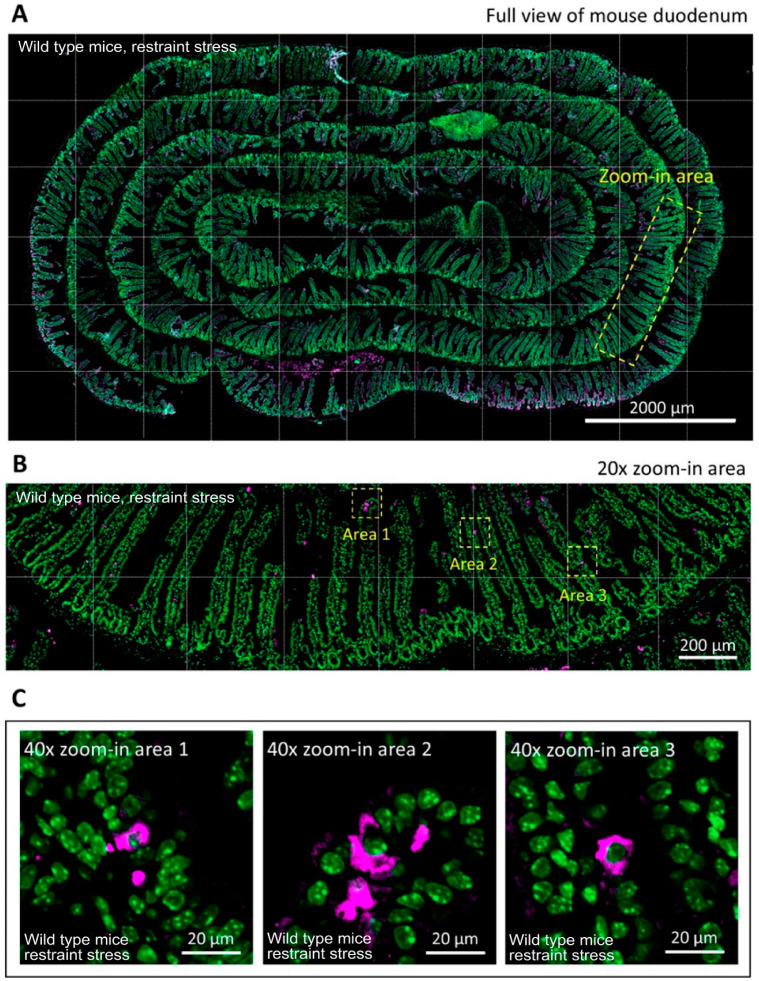
Confocal microscopy of IHC activated-form caspase 3 staining. A tissue section IHC of a Swiss-rolled duodenal sample was stained with a fluorescence-labeled anti-activated-form caspase 3 antibody. (**A**) A representative image at low magnification is presented. (**B**) The yellow boxed zoomed-in area in (**A**) is enlarged as a higher magnification view. (**C**) Areas 1, 2, and 3 in (**B**) are further enlarged to observe the detailed expression patterns of activated-form caspase 3 in the villi of duodenal tissue. Scale bars: 2000 μm (**A**), 200 μm (**B**), and 20 μm (**C**).

**Figure 10:**
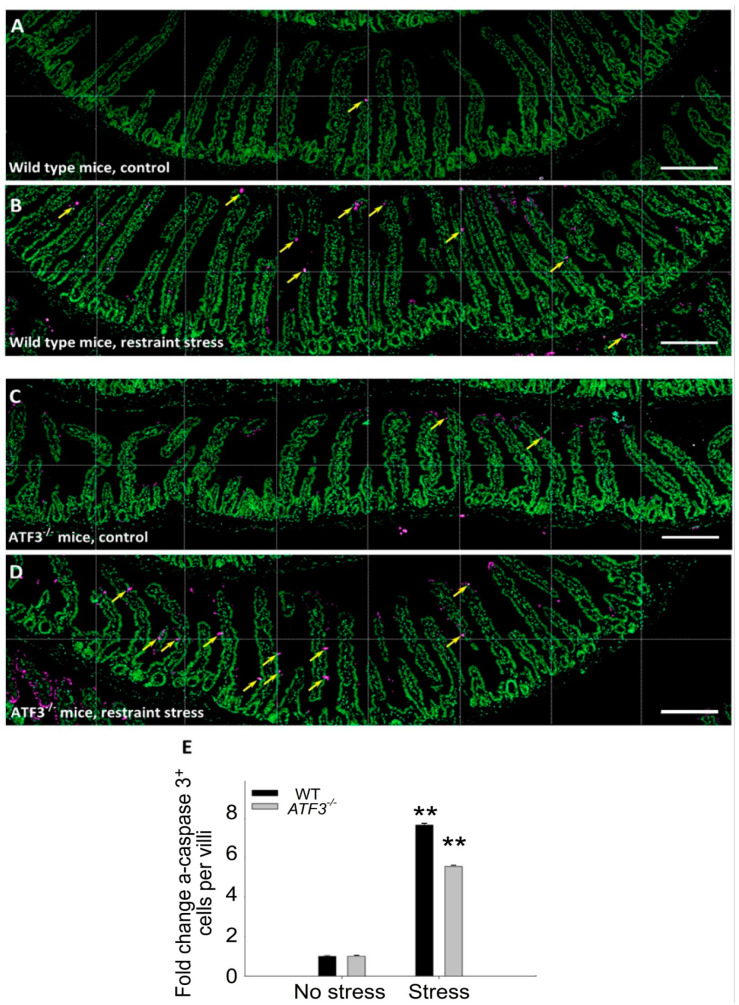
Relative activated-form caspase 3 staining signals in WT and *ATF3^−/^^−^* mice after stress. The WT mice (**A**,**B**) was compared to *ATF3^−/^^−^* mice (**C**,**D**) without (**A**,**C**) and with (**B**,**D**) restraint stress. (**E**) The quantitative results of activated-form caspase-3^+^ (a-caspase 3^+^) apoptotic epithelial cells and villi were obtained from three images at various areas. ** *p* < 0.01 vs. the respective no stress groups. Scale bars: 200 μm (**A**–**D**). Error bars show the standard deviation.

Using annexin V, PI, and CD326 triple staining and flow cytometry, we determined that stress induced markedly higher levels of duodenal epithelial cell death (CD326^+^/PI^+^/annexin V^+^ cell population) in both WT and *ATF3^−/−^* mice than in the no stress groups (Figure 8A–J). Consistent with this, the duodenal epithelial cells of the WT and *ATF3^−/^^−^* mice also displayed considerably higher levels of activated and cleaved-form caspase 3 signals after stress (Figure 8K–P) than the control mice. These results suggest that hours of restraint stress are sufficient to induce the apoptotic cell death of duodenal epithelial cells in mice. Confocal microscopy and IHC analyses were performed to verify this result. Consistently, the IHC data of Swiss-rolled duodenal tissue samples revealed that restraint stress induced activated-form caspase-3-positive intestinal cells specifically in the villi areas (Figure 9). Compared with the control groups, the activated-form caspase-3-positive intestinal cells were markedly increased after stress in the WT and *ATF3^−/^^−^* mice (Figure 10). These results suggest that, in addition to the dysregulation of tight junction proteins, duodenal epithelial cell death is also involved in stress-induced ulcer-like injuries.

## 4. Discussion

Peptic ulcer is a disruption of the mucosal surfaces in the inner lining of the GI tract. The primary causes of a peptic ulcer include bacteria *Helicobacter pylori*, nonsteroidal anti-inflammatory drugs (NSAIDs), tobacco smoking, genetic diseases, and physiological and psychological stresses [61,62,63,64,65,66]. PPI therapy is a primary treatment for peptic ulcers, but it may cause adverse effects [67]. Compared with the peptic ulcers of other causes, psychological stress-induced peptic ulcers are rarely studied. Evidence has revealed that, independent of *H. pylori* infection or receiving a therapy of NSAIDs, psychological stress markedly increases the risk of peptic ulcers in participants without a prior history of peptic ulcers [64,68]. Additionally, in the animal studies, the methods of analyzing GI injuries were primarily available after sacrifice [69,70,71]. As a result, a favorable live animal model for the treatments of peptic ulcers is required. In this study, we developed the novel orally administered Evans blue live mouse model, which can be repeatedly monitored and analyzed at various time points by measuring the plasma Evans blue levels without sacrificing the animal.

Evans blue dye has a long history of being used as a biological and clinical agent [72]. After intravenous injection, Evans blue, an azo dye with a high affinity for serum albumin, can bind to the plasma protein albumin to form a 69-kDa tracer, a molecular-sized protein not normally able to penetrate viable cells and peripheral tissues unless vascular leakage or tissue injuries occur [73]. As a result, Evans blue dye is widely used to measure tissue injuries [35,42,74,75,76]. This is likely the reason that Evans blue has previously only been used in analyses of gut leakage using proximal–distal-ligated intestine samples that were isolated from the sacrificed animal [77]; the leakage levels of dead, not live intestinal tissue were recorded. Accordingly, the best time points for the analysis of other cellular and biochemical parameters such as RNA, protein expression, and the cell death status may not be easily available. We demonstrated that the oral administration of Evans blue is a feasible new method for tracking intestinal leakage in live animals. For the development of potential clinical use, a patient-friendly lower-toxicity version of Evans blue or other GI-nonabsorbable highly sensitive tracking dyes are desired.

In this present study, PPI treatments can rescue stress-induced GI leakage in mice. This suggests that the GI pH homeostasis is disrupted by acute restraint stress. The proton pump is an H^+^/K^+^ ATPase located in the parietal cell responsible for acid secretion into the gastric lumen [78]. PPIs are weak bases that accumulate in the acidic space, inhibiting the proton pump to suppress acid secretion [78]. In normal conditions, the gut epithelium in acid-secreting gastric mucosa exports HCO_3_ to form a mucus–bicarbonate barrier and maintain a neutral pH at the apical cell surfaces [79]. In this present study, we found that mouse duodenal tissue becomes more acidic after restraint stress, which is associated with downregulated tight junction expression and increased cell death of gut epithelial cells. This suggests a dysregulation of mucus–bicarbonate barrier function occurs after restraint stress and likely the reason that PPI treatments can rescue the stress-increased permeability of GI. Additionally, ATF3 deficiency exacerbates the GI leakage, suggesting ATF3 is directly or indirectly involved in this stress-induced pathophysiology.

As a stress-induced transcription factor, ATF3 plays critical roles in the regulation of cell survival, metabolism, immunity, and oncogenesis [12,80]. Various cellular stress signals, such as endoplasmic reticulum stress, cytokines, chemokines, and bacterial lipopolysaccharide, are associated with ATF3 induction [12]. Although the mechanism remains elusive, the emerging roles of ATF3 in the regulation of GI homeostasis has been revealed. For example, ATF3 is critical in the regulation of intestinal regeneration [16], intestinal immunity [13,15], and maintaining healthy intestinal microbiota [14]. The role of ATF3 in stress ulcers, however, remains elusive.

This report demonstrates the pathogenic alternations of an acute stress ulcer in a mouse model. ATF3 deficiency greatly exacerbates stress-induced GI damages, suggesting a protective role of ATF3 toward the stress ulcer. The literature revealed that long-term chronic stress can lead to dysbiosis and GI tract dysfunction [81,82] and may further exacerbate neurodegenerative and psychological diseases [83,84]. In experimental colitis, ATF3 modulated the gut epithelial barrier functions and protected against inflammation-associated injuries in mice [85]. However, the role of ATF3 in GI cellular stress and injury in acute stress ulcers is less discussed. Similar to the gut dysbiosis observed in inflammatory GI diseases [86,87], gut microbiota become proinflammatory after long-term restraint stress [24,88]. The mechanism of pathophysiological changes at the early phase of stress-induced GI injuries is theoretically crucial for the development of therapeutic approaches against these GI idiopathic inflammatory diseases, in which ATF3 likely plays a role in the maintenance of homeostasis.

In this present mouse model, restraint stress induced ATF3 expression in intestinal epithelial cells of mice. ATF3 deficiency exacerbated the stress-induced GI permeability, which is associated with a disrupted expression of epithelial tight junction. In general, tight junction gene expression plays a protective role in the GI system, while tight junction disruption indicates GI injuries [89,90,91]. This suggests a protective role of ATF3 in the rescue of stress-induced damage. The literature has reported a protective role of ATF3 on wide-spectrum stress signal-induced cell deaths in numerous cell types. For example, in the vascular system, ATF3 increases the survival rates of vascular smooth muscle cells [92] and protects endothelial cells from tumor necrosis factor α-induced cell death [93]. In the neuronal system, ATF3 plays a pro-survival role in ganglion cells and neurons [94]. Consistently, ATF3 protects against doxorubicin-induced apoptosis in cardiac myocytes [95] and stress-induced cell death in renal cells, β-cells [96], and keloid fibroblasts [97]. The collective evidence suggests that ATF3 plays a protective role in cell-stressed conditions. Therefore, observing the GI epithelial cell protective effects of *ATF3* in stress ulcers is reasonable. It is noteworthy that ATF3 also plays vital roles in the nervous system [94]. Consequently, ATF3 may also be involved in the regulation of GI permeability at the “brain” side of the gut–brain axis. The contribution of neuronal ATF3 regulation to GI leakage is worth further investigation.

## 5. Conclusions

Using this new Evans blue-restraint stress mouse model, we determined that the dysregulation of tight junction expression and apoptotic epithelial cell death is involved in stress-induced ulcer-like damages. An ATF3 deficiency exacerbates stress-induced GI leakages, which are associated with the abnormal regulation of epithelial tight junction expressions. These results suggest that, as a native cellular protective pathway in the GI system, ATF3 could be a molecular target for managing psychological stress-induced GI tract diseases. Since long-term stress can lead to GI dysbiosis and inflammatory diseases, these findings could be useful for the development of therapeutic approaches against acute stress ulcers and stress-associated chronic inflammation.

## Data Availability

The data presented in this study are available in the article and the supplementary material.

## Supplementary Materials

The following are available online at https://www.mdpi.com/article/10.3390/cells10123530/s1: Figure S1: Stress-induced GI abnormalities. Table S1: List of primers used in this report.
