# Supplementary material for: Activating Transcription Factor 3 Protects against Restraint Stress-Induced Gastrointestinal Injury in Mice
Source: Cells. 2021 Dec 14;10(12):3530. doi: 10.3390/cells10123530 (PMC8700235; doi:10.3390/cells10123530)
Supplement: Supplementary file 1 [file cells-10-03530-s001.zip › cells-1474773-supplementary.pdf]

Supplemental information for:

Activating transcription factor 3 protects against restraint stress-induced gastrointestinal injury in mice

Dun-Jie Chuang[1], Subhashree Pethaperumal[1], Bijaya Siwakoti[1], Hung-Jen Chien[2], Ching-Feng Cheng[3,4], Shih-Che Hung[5], Te-Sheng Lien[1], Der-Shan Sun[1,5], and Hsin-Hou Chang,[1, 5]*

[1] Department of Molecular Biology and Human Genetics, Tzu-Chi University, Hualien, Taiwan.

[2] Institute of Biotechnology, National Tsing Hua University, Hsinchu, Taiwan

[3] Department of Pediatrics, Taipei Tzu Chi Hospital, Buddhist Tzu Chi Medical Foundation, Taipei, Taiwan

[4] Institute of Biomedical Sciences, Academia Sinica, Taipei, Taiwan

[5] Institute of Medical Sciences, Tzu-Chi University, Hualien, Taiwan.

Running title: ATF3 rescues stress ulcer in mice

Key words: Activating transcription factor 3, ATF3, gastrointestinal tract, intestinal leakage, tight junction, cell death, gut epithelial cell, gastric ulcer

* Correspondence, Hsin-Hou Chang, E-mail: hhchang@mail.tcu.edu.tw

Figure S1 and Table S1

Figure S1

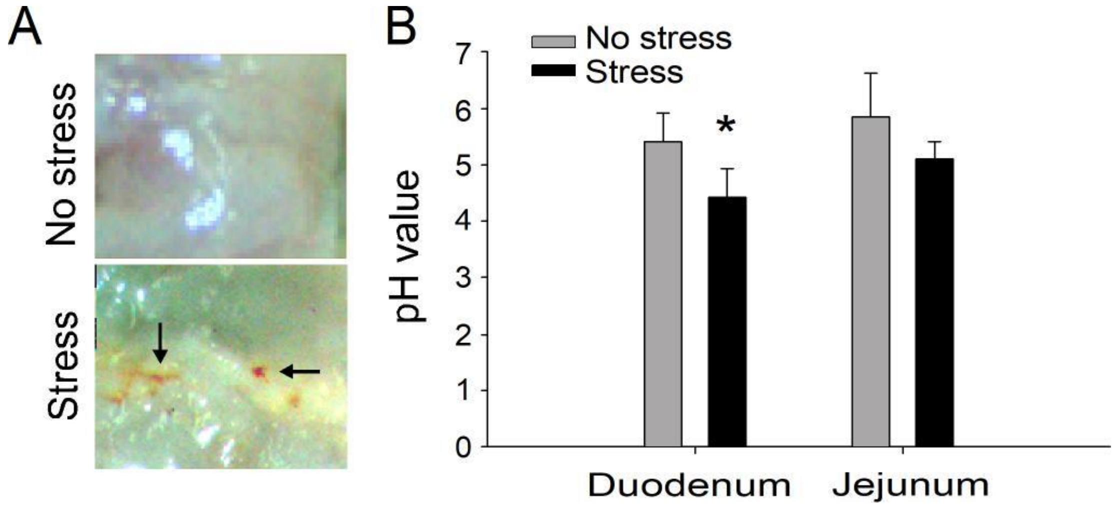

Figure S1. Stress induced GI abnormalities. (A) Representative image of hemorrhage lesions observed in GI tissue samples of stressed mice (arrows indicated regions), but not observed in tissue samples of no stress controls. (B) Duodenal tissue samples from 9-h stressed mice displayed lower pH value as compared to the no stress controls. By contrast, the analysis of the jejunum tissue samples does not display differences. n = 12, * $P < 0.05$, vs. no stress control groups.

Table S1

List of primers used in this report

Gene symbol	Forward primer (5'→3')	Reverse primer (5'→3')	Product size (bp)	Temperature (°C)
**GAPDH**	TCAACAGCAACTCCCACTCTTCCA	ACCCTGTTGCTGTAGCCGTATTCA	115	55
**ZO-1**	AGGACACCAAAGCATGTGAG	GGCATTCCTGCTGGTTACA	87	55
**Cldn3**	CCACTACCAGCAGTCGATGA	GGAAGGGCGAGGTTTCTTTG	211	60
**Jam3**	GGCTGCGACTTCGACTGTA	GAGATTCACTGCCTCTATCATGC	86	55
**ATF3**	AGTGACAGCATGAGCCCTCT	GCAGCACTGACCTGATCAAA	179	60
